# Facile fabrication of CoAl-LDH nanosheets for efficient rhodamine B degradation *via* peroxymonosulfate activation†

**DOI:** 10.1039/d3ra04575g

**Published:** 2023-10-10

**Authors:** Hui Fui, Shumin Gao, Xinran Ma, Yiping Huang

**Affiliations:** a School of Chemical and Environmental Engineering, Wuhan Polytechnic University Wuhan 430023 Hubei Province PR China feihui509@163.com

## Abstract

Layered double hydroxides (LDHs) have been extensively investigated as promising peroxymonosulfate (PMS) activators for the degradation of organic pollutants. However, bulk LDHs synthesized using conventional methods possess a closely stacked layered structure, which seriously blocks active sites and yields low intrinsic activity. In this study, we exfoliated bulk CoAl-LDHs to fabricate CoAl-LDH nanosheets by alkali-etching and Ostwald ripening *via* a simple hydrothermal process in a KOH solution. The exfoliated LDHs possessed the typical nanosheet structure with more exposed active sites for PMS activation, and hence, boosted the degradation of the pollutants. CoAl-1 exhibited an outstanding catalytic performance as the PMS activator for rhodamine B (RhB) degradation with the apparent rate constant of 0.1687 min^−1^, which was about 3.63 and 5.02 times higher than that of commercial nano-Co_3_O_4_ and bulk CoAl-LDH, respectively. The maximum RhB degradation of 93.1% was achieved at the optimal reaction conditions: catalyst dose 0.1 g L^−1^, PMS concentration 0.3 mM, pH 7, and temperature 298 K. Further analysis of RhB degradation mechanism illustrated that singlet oxygen (^1^O_2_) dominated RhB degradation in the CoAl-1/PMS system, while ˙OH, ˙O_2_^−^, and ˙SO_4_^−^ may mainly serve as the intermediates for the generation of ^1^O_2_ and were indirectly involved in the degradation. This study provides a promising strategy for developing two-dimensional LDH nanosheets for wastewater remediation.

## Abstract 2

An exfoliation strategy of bulk CoAl-LDHs was developed to afford two-dimensional nanosheets by alkali-etching and Ostwald ripening, which exhibited superior catalytic activity compared with bulk CoAl-LDHs and commercial nano-Co__3__O__4__.

## Graphical abstract

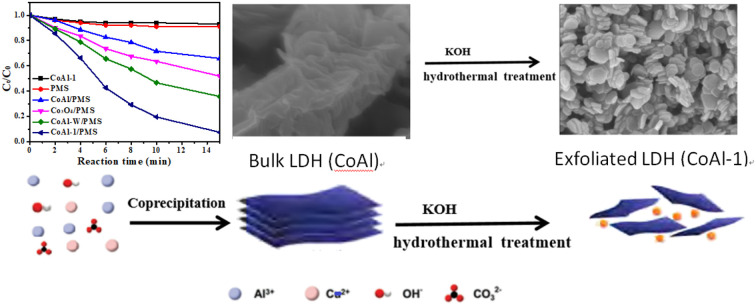

## 1. Introduction

The intensification of organic pollutants, such as pesticides, pharmaceuticals, personal care products, and organic dyes, in water seriously threatens human health and has acquired tremendous attention.^1–5^ Numerous technologies (*e.g.*, adsorption method, membrane separation, bio-degradation, and advanced oxidation processes (AOPs)) have been undertaken to eliminate these organic pollutants from water.^6–9^ Among them, persulfate-based advanced oxidation processes have been recognized as a highly promising approach where the activation of persulfates (peroxymonosulfate (PMS) and peroxydisulfate (PDS)) can produce a series of powerful species (*e.g.*, ˙SO_4_^−^, ˙OH, and ^1^O_2_), and these species can degrade or mineralize organic contaminants.^10^ In general, PMS can be more easily activated than PDS in homogeneous or heterogeneous systems by means of various methods (*e.g.*, thermal, ultraviolet, alkaline, transition metals, and carbon materials).^11–15^ However, transition metals (*e.g.*, Co, Mn, and Cr) are a feasible way to activate PMS.^16,17^ Unfortunately, transition metal ions used in the homogeneous system are difficult to recover from the reaction solution and inevitably cause secondary pollution. Thus, the construction of high-performance heterogeneous catalysts as PMS activators is highly desired.

As typical transition metal-based materials, layered double hydroxides (LDHs) with positively charged host layers and intercalated charge balancing anions^18–20^ are regarded as promising PMS activators for organic pollutant degradation because of their highly controllable layered structure and metal composition (*e.g.*, interlayer anions, number of layers, metal species, and metal ratios).^21–23^ However, LDH layers tend to stack or even form bulk LDHs with the closely stacked layered structure, which blocks active sites and leads to low catalytic activities.^24^ In this regard, the exfoliation of bulk LDHs into LDH nanosheets is an effective strategy to increase the surface area and exposed active sites. Thus, numerous strategies have been developed to exfoliate the pristine bulk LDH into ultrathin nanosheets. Zhang *et al.*^25^ reported a two-step strategy to exfoliate bulk LDHs using zwitterionic surfactants, in which zwitterionic surfactants were first inserted into the LDH interlayers by ion exchange, and then the surfactant-intercalated LDHs were exfoliated to obtain LDH nanosheets in an organic solvent. Yu *et al.*^26^ prepared LDH nanosheets by exfoliating bulk LDHs directly in a formamide solution without the pre-synthesis of surfactant-intercalated LDHs. Nevertheless, organic solvents used in the above exfoliation process tend to attach on the surface of the obtained LDH nanosheets, thus blocking the exposed active sites.^27,28^ Recently, Chen *et al.*^29^ presented a novel exfoliation method for NiFe LDHs by Ostwald ripening *via* a simple hydrothermal treatment without using extra reagents or surfactants. Nevertheless, the Ostwald ripening driven exfoliation usually takes a long time. Therefore, an efficient strategy needs to be explored for fabricating clean LDH nanosheets by exfoliating the bulk LDHs.

Herein, we report an exfoliation strategy of bulk CoAl-LDHs to afford two-dimensional nanosheets by alkali-etching and Ostwald ripening *via* a facile hydrothermal treatment in KOH solution. The best samples exhibited superior catalytic activity for RhB degradation compared with bulk CoAl-LDHs and commercial nano-Co_3_O_4_. Moreover, the effect of the reaction parameters (*e.g.*, pH, temperature, catalyst dosage, and PMS concentration) on RhB degradation was systematically investigated. Further analysis of the RhB degradation mechanism in the CoAl-1/PMS system was performed using electron paramagnetic resonance (EPR) and a series of scavenger experiments. This study provides a promising strategy for developing thin-layer LDHs for the removal of organic pollutants.

## 2. Experimental section

### 2.1 Materials and reagents

Cobaltous nitrate hexahydrate (Co(NO_3_)_2_·6H_2_O), sulfuric acid (H_2_SO_4_), sodium carbonate (Na_2_CO_3_), aluminum nitrate nonahydrate (Al(NO_3_)_3_·9H_2_O), sodium thiosulfate (Na_2_S_2_O_3_), furfuryl alcohol (FFA), 5,5-dimethyl-1-pyrroline *N*-oxide (DMPO), rhodamine B (RhB), potassium peroxymonosulfate (PMS), *tert*-butyl alcohol (TBA), 2,2,6,6-tetramethyl-4-piperidone (TMP), *p*-benzoquinone (*p*-BQ), methanol (MeOH), and commercial nano-Co_3_O_4_ (30 nm, 99.5%) were supplied by Aladdin Company, China and used as received. Deionized water was used in all the experiments.

### 2.2 Catalyst preparation

CoAl-LDHs were synthesized using a conventional coprecipitation method. Typically, solution A was pre-prepared by adding Co(NO_3_)_2_·6H_2_O (0.08 mol), Al(NO_3_)_3_·9H_2_O (0.04 mol) to deionized water (80 mL). Then, 80 mL of solution B containing NaOH (0.24 mol) and Na_2_CO_3_ (0.2 mol) was added dropwise to solution A under magnetic stirring. After continuously stirring for 0.5 h, the resulting suspensions were aged at 60 °C for 24 h in a water bath. Finally, the precipitate was washed several times with deionized water and ethanol and then dried at 60 °C overnight in a vacuum to obtain CoAl-LDHs.

CoAl-LDH nanosheets were synthesized by exfoliating the CoAl-LDHs precursor through a simple hydrothermal treatment in KOH aqueous solution. Briefly, 300 mg CoAl-LDHs was added to a 100 mL Teflon-lined autoclave with 80 mL of potassium hydroxide aqueous solution (0.5 M). The autoclave was then heated to 120 °C and maintained for different times. Afterward, the resulting product was rinsed with deionized water and ethanol several times and dried at 60 °C under vacuum for 24 h to obtain exfoliated CoAl-LDH. According to different hydrothermal times (0.5, 1, and 2 h), the as-prepared exfoliated CoAl-LDH samples were labeled as CoAl-0.5, CoAl-1, and CoAl-2, respectively. For comparison, the samples (denoted as CoAl-*W*) were fabricated under the same hydrothermal conditions for 1 h without the addition of KOH.

### 2.3 Catalyst characterization

The phase structures of the as-fabricated catalyst were analyzed by X-ray powder diffraction (XRD) on a Bruker D8 Advance powder diffractometer using Cu Kα radiation (*λ* = 0.154 nm). The morphology was examined using a Zeiss GeminiSEM 300 scanning electron microscope (SEM). Elemental mappings were performed by energy-dispersive X-ray spectroscopy (EDS, Oxford X-MAX) on the SEM tool. X-ray photoelectron spectroscopy (XPS) was conducted on a Thermo Scientific Escalab 250Xi spectrometer with an Al Kα anode, and the binding energy was standardized with respect to the residual C 1s peak.

### 2.4 Experimental procedures

PMS activation performance of the as-fabricated catalyst was evaluated using RhB as a probing molecule. Typically, 10 mg catalyst was added to 100 mL of RhB solution (80 mg L^−1^) under mechanical stirring. Afterward, 0.3 mM PMS was added to the suspension to initiate the reaction. 2 mL samples were withdrawn at regular intervals, quenched with Na_2_S_2_O_3_ solution, and filtered using a 0.22 μm filter. The concentrations of RhB were analyzed using a UV-vis spectrophotometer (Lambda 650). 0.1 M NaOH or 0.1 M H_2_SO_4_ was used to adjust the initial pH values. To explore the mechanism of RhB degradation, electron paramagnetic resonance (EPR) was performed on a Bruker A300 spectrometer to identify the presence of reactive species. A series of quenching experiments were conducted using TBA, FFA, methanol, and *p*-BQ as radical scavengers to investigate the effect of these species on RhB degradation.

## 3. Results and discussion

### 3.1 Synthesis and characterization of catalysts

**Fig. 1:**
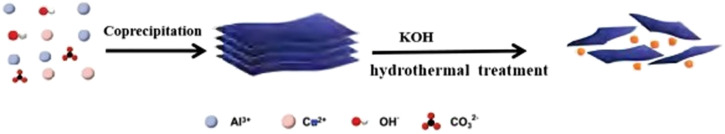
Schematic illustration of the synthetic route of CoAl-LDH nanosheets. (Al/Co atomic ratio used in the coprecipitation was 1 : 2, hydrothermal temperature was 180 °C, and KOH concentration was 0.5 M).

**Fig. 2:**
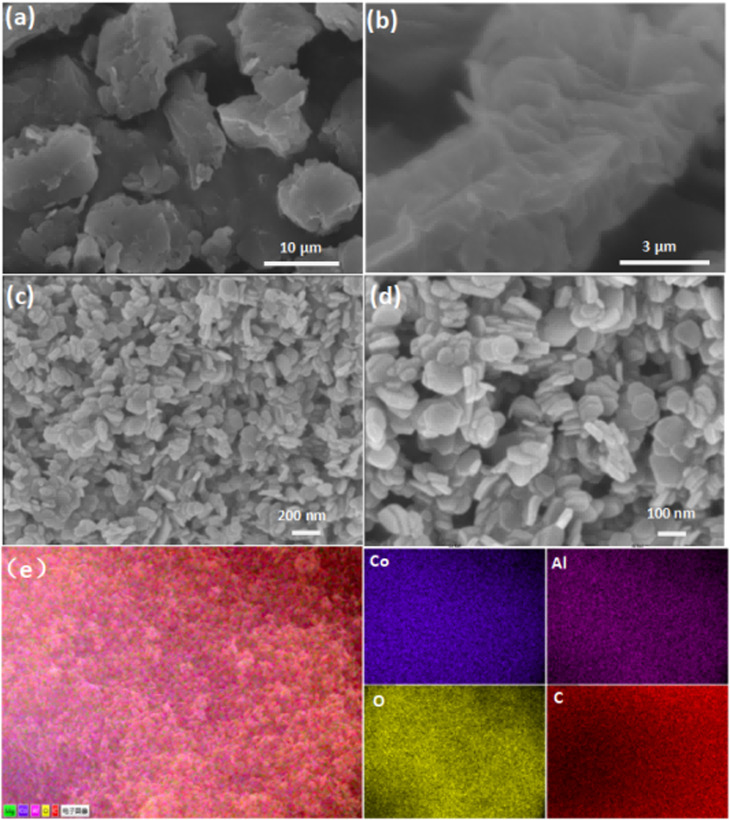
Low and high magnification SEM images of CoAl-LDH (a and b) and CoAl-1 (c and d); EDS mapping images (e) of CoAl-1.

As illustrated in Fig. 1, the synthesis of CoAl-LDH nanosheets involved the pre-synthesis of the CoAl-LDH precursor, followed by exfoliation of the as-prepared precursor. The morphology of bulk CoAl-LDH and the typical exfoliated samples (CoAl-1) were characterized by SEM. As shown in Fig. 2a and b, the bulk CoAl-LDH exhibited an agglomerated plate morphology with a lateral size of 8.0–10.0 μm and an average thickness of 5.0–7.0 μm, which indicated that the pristine bulk LDHs comprised of multitudinous closely stacked LDH layers. After the exfoliation process, the morphology of CoAl-1 was significantly different from that of bulk CoAl-LDHs. As shown in Fig. 2c and d, CoAl-1 displayed hexagonal nanosheet morphology. The average thickness decreased to about 7.5 nm, and the number of stacked layers also decreased to about 9 or 10 layers (since a single LDH layer has a thickness of about 0.8 nm (ref. 30)). Meanwhile, EDS mapping analyses (Fig. 2e) confirmed a homogeneous distribution of Co, Al, O, and C on the near surface of CoAl-1. Furthermore, the Co/Al molar ratio (2.1 : 1.0) in CoAl-1 was higher than that in the synthesis mixture (2.0 : 1.0), indicating that Al vacancies were introduced by the exfoliation of bulk LDHs. The study on the exfoliation of other bulk materials also showed similar results.^31^

**Fig. 3:**
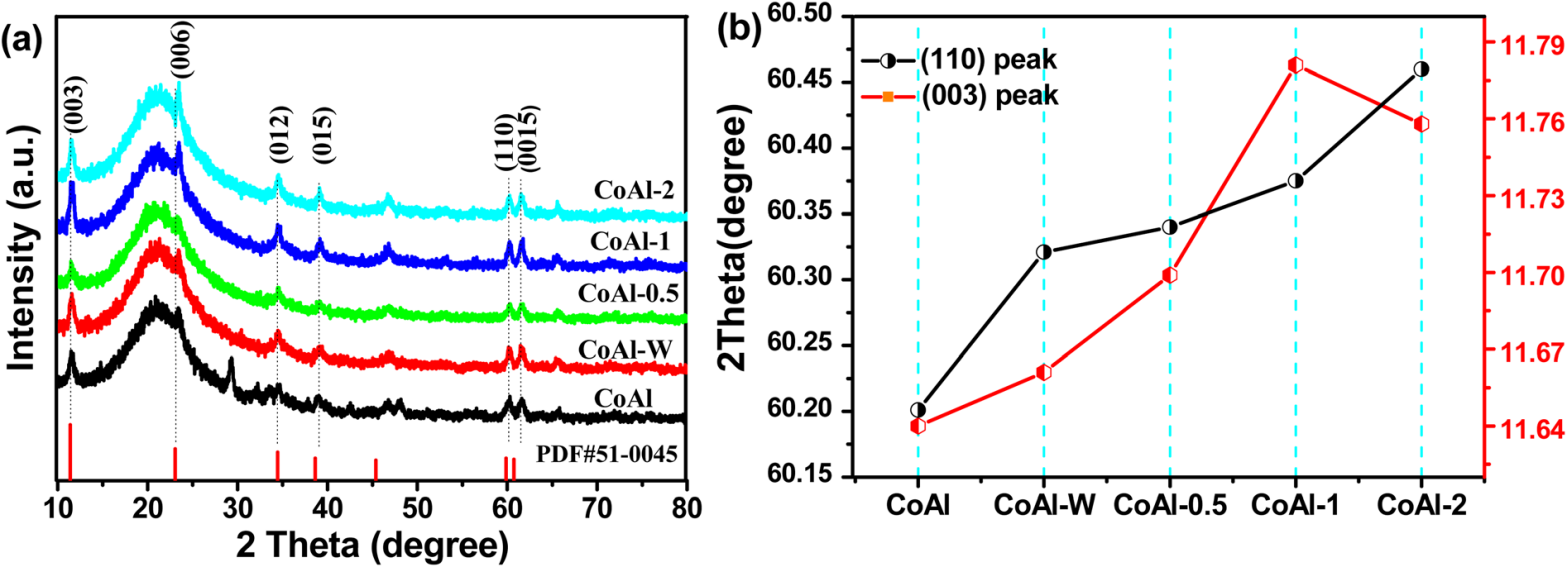
(a) XRD patterns of different samples; (b) 2-theta angle for the (003) and (110) peak.

The crystal structures of bulk CoAl-LDH and the exfoliated CoAl-LDH were studied by XRD. As shown in Fig. 3a, each sample manifested the characteristic diffraction peak of a rhombohedral LDH structure,^32^ in which the peaks at 11.66, 23.57, 34.94, 39.42, 60.32, 60.72° were assigned to the (003), (006), (012), (015), (110) and (0015) crystal planes, respectively, of CoAl-LDH (PDF51-0045).^33^ Note that the diffraction peaks at 19.30°, and 20.53° could be indexed to Co-LDH (PDF30-0443) and CoOOH (PDF07-0169), respectively. This phenomenon could be attributed to the fact that these products could be generated and their crystal structures were well preserved during the fabrication of CoAl-LDHs. After the exfoliating process, the (003) peak (Fig. 3b) for exfoliated CoAl-LDHs material was located at lower 2*θ* angles compared with the corresponding reflection for bulk CoAl-LDH, which might be attributed to smaller crystal size and a higher degree of disorder, thereby, lowering the 2*θ* angle of the (003) reflection.^34^ Furthermore, the (110) peak (Fig. 3b) shifted to higher 2*θ* angles as the exfoliation time increased, suggesting an in-plane compressive strain was introduced in the LDH nanosheets. The compressive strain was mainly due to the loss of aluminum and oxygen ions from the host layers during the exfoliation process. Overall, the above results proved that CoAl-LDH nanosheets were successfully prepared by exfoliating bulk LDH *via* hydrothermal treatment in a KOH solution.

**Fig. 4:**
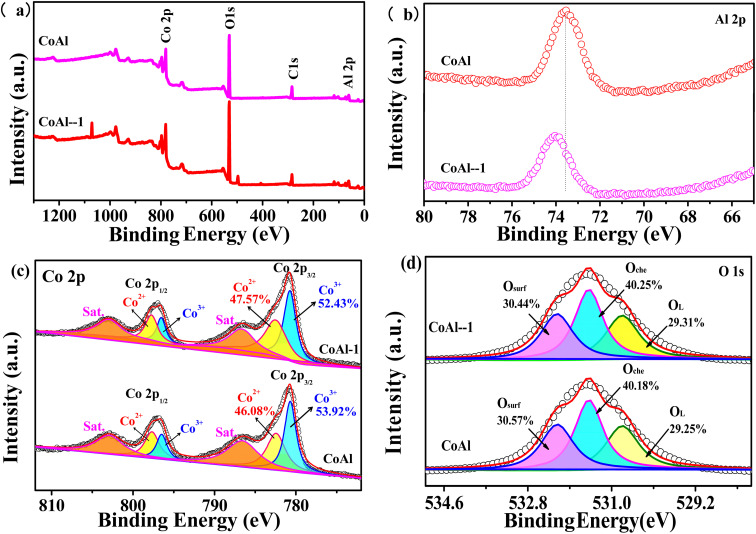
XPS spectra of different samples. (a) Survey; (b) Al 1s; (c) Co 2p; (d) O 1s.

The surface chemical bonding states of CoAl-LDHs and CoAl-1 were characterized by XPS measurements. The survey spectrum in Fig. 4a again confirms the presence of Co, Al, O, and C. In the Al 2p spectra (Fig. 4b), the main peak was corresponding to Al^3+^ species. However, the peak intensity of CoAl-1 decreased, and the peak positions shifted negatively compared with that of CoAl LDHs, which might be attributed to the introduction of Al vacancies during the exfoliation process, thus creating different chemical/electron environments for Al atoms. It was noted that Co 2p XPS spectra and O 1s spectra showed little difference in peak shapes and positions for the two samples. In the Co 2p spectra (Fig. 4c), the two peaks corresponding to Co 2p_3/2_ and 2p_1/2_, are centered at 781.1 and 797.0 eV, respectively. The Co 2p_3/2_ peak can be fitted by Lorentzian–Gaussian, and the three fitted peaks corresponding to Co^2+^, Co^3+^, and satellite peaks were located at 782.4, 780.5, and 802.1 eV, respectively.^35^ However, the Co^2+^/Co^3+^ molar ratio in CoAl-1 was calculated to be 0.524 from their fitted peak area ratio, which is higher than that (0.476) of CoAl-LDHs, implying partial Co^3+^ was reduced to Co^2+^ after the exfoliation process.

As shown in Fig. 4d, the O 1s spectra were fitted into three peaks, which were located at 529.1, 530.6, and 532.1 eV, representing lattice oxygen (O^2−^), absorbed oxygen (O_2_^2−^ and O_2_^−^), and hydroxyl groups (OH^−^), respectively. The peak area of the adsorbed oxygen represents the oxygen vacancy content. According to their fitted peak area ratio, the oxygen vacancy content was estimated to be 40.3% for CoAl-1 and 40.2% for CoAl-LDHs. According to the XPS spectrum analysis, a more plausible explanation is that Al vacancies might introduce oxygen vacancies into the LDH during the exfoliation process, and subsequently, the localized electrons in the oxygen vacancies would lead to the reduction of Co^3+^ to Co^2+^. More Co^2+^ and oxygen vacancies were the excellent active sites for the activation of PMS and hence improved the degradation of the pollutant.

### 3.2 Catalytic performance evaluation

**Fig. 5:**
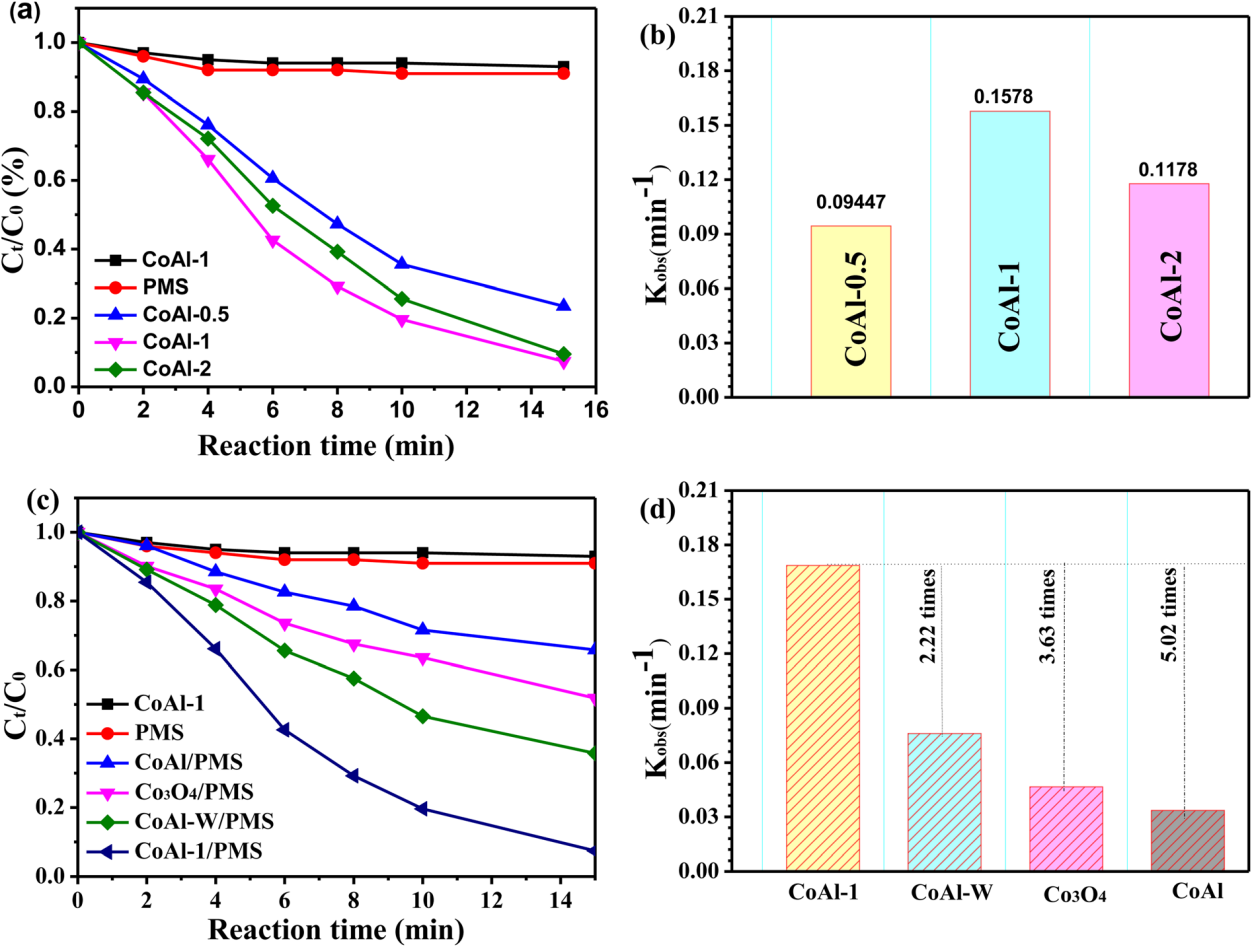
(a) Degradation kinetics and (b) rate constant of RhB for the exfoliated CoAl-LDH; (c) degradation kinetics and (d) rate constant of RhB for bulk CoAl-LDH and CoAl-1. Experiment conditions: pH = 7, *T* = 298 K, [RhB] = 80 mg L^−1^, [catalyst] = 100 mg L^−1^, [PMS] = 0.3. mM.

The catalytic activities of the as-synthesized catalysts were investigated by activating PMS for RhB degradation. As shown in Fig. 5a, only a very small amount of RhB was removed when PMS or each catalyst was presented separately, suggesting that neither the single chemical oxidation nor the adsorption process could efficiently remove RhB. However, RhB degradation efficiency was significantly enhanced to 76.6% within 15 min when CoAl-0.5 and PMS were simultaneously introduced into the system. The exfoliated CoAl-LDH prepared with different exfoliation times exhibited different catalytic activities for RhB degradation. The highest RhB removal efficiency (93.1%) was achieved when CoAl-1 was used as the PMS activator. However, further enhancement was not observed, instead a slight decrease in removal rate occurred in the CoAl-3/PMS system. Combining the results of the XRD analyses, we have demonstrated that the activity order of the exfoliated CoAl-LDH was similar to the 2*θ* angle of the (003) reflection, implying that the different catalytic performances of the three catalysts might be ascribed to the crystal size.

Fig. 5b shows the catalytic activities of bulk CoAl-LDH and CoAl-1. CoAl-1 had excellent RhB degradation efficiency (93.1%) within 15 min, which was almost 2.7 times higher than that of bulk CoAl-LDH (34.2%). For further comparison, we evaluated the catalytic degradation of RhB with CoAl-*W* and nano-Co_3_O_4_ under the same reaction conditions, and RhB removal was only 64.2% for CoAl-*W*, and 48.2% for nano-Co_3_O_4_. In addition, based on the pseudo-first-order kinetics model,^36^ the degradation rate constant (*k*) in different systems was calculated. As shown in Fig. 3b, the rate constant (*k*) of the CoAl-1/PMS system was about 0.1687 min^−1^, which was about 2.22, 3.63, and 5.02 times higher than that of CoAl-*W*, nano-Co_3_O_4_ and bulk CoAl-LDH, respectively. These results indicated the obvious superiority of CoAl-1 during the catalytic oxidation over those of the other catalysts under the same reaction conditions, which could be attributed to the fact that CoAl-1 possessed the typical 2-dimensional structure. As has been previously reported,^37^ RhB is mainly degraded *via* PMS activation on the surface of the catalyst. PMS activation starts with the adsorption of PMS on the catalyst surface. Next, the charge transfer between catalysts and PMS generates SO_4_˙^−^, which ultimately leads to the degradation of organic pollutants. As a result, the typical 2-dimensional structure of CoAl-1 provides a large specific surface area and more exposed active sites, which facilitates the contact of the catalysts with PMS and organic pollutant molecules, thus further enhancing the adsorption and catalytic performance.

### 3.3 Effects of the reaction parameters on RhB degradation

**Fig. 6:**
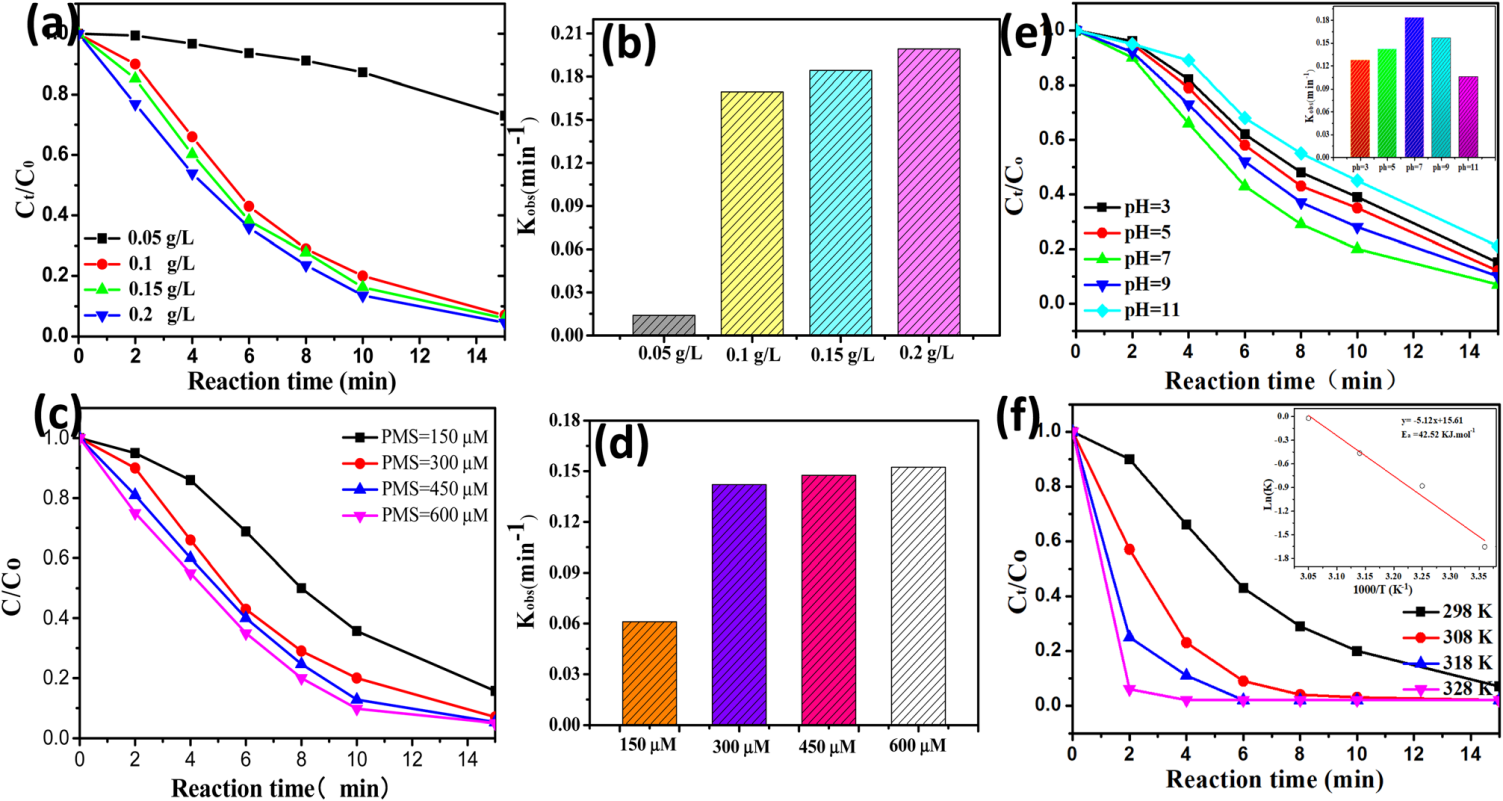
Effects of (a) catalyst dose, (c) PMS concentration, (e) initial solution pH, (f) solution temperature on RhB degradation efficiencies in CoAl-1/PMS system, and the corresponding fitting kinetic parameters of (b) catalyst dose and (d) PMS concentration. [Reaction conditions: [RhB] = 80 mg L^−1^, [PMS] = 0.3 mM, [catalyst] = 100 mg L^−1^, *T* = 298 K and pH = 7.]

Some key reaction parameters (*e.g.*, initial solution pH, reaction temperature, catalyst dosage, and PMS concentration) of the CoAl-1/PMS system were investigated and optimized. As shown in Fig. 6a and b, the degradation efficiency of RhB was significantly increased from 26.9% to 93.1% within 15 min when the catalyst dosage was increased from 0.05 to 0.1 g L^−1^, and the corresponding degradation rate constants (*k*) was obviously increased from 0.01397 to 0.1687. It could be attributed to the fact that increasing the catalyst dosage resulted in more active sites for PMS activation and generated more powerful radical species, thus accelerating RhB degradation. Nevertheless, when the catalyst dosage was further increased from 0.1 to 0.2 g L^−1^, the improved RhB degradation was not observed. This phenomenon could be attributed to the fact that the free radicals were generated to a maximum degree from the fixed PMS. In addition, an overabundance of the catalyst would generate excess radicals, and the scavenging effect of the excessive radical species would also suppress the catalytic degradation of RhB.^38^ Therefore, the catalyst dosage of 0.1 g L^−1^ was selected as the proper dosage.

As shown in Fig. 6c and d, the PMS concentration had a positive but limited effect on RhB degradation. The RhB degradation efficiency was gradually increased from 84.3% to 93.1% within 15 min when the PMS concentration was increased from 0.15 to 0.3 mM. Nevertheless, the RhB degradation efficiency only exhibited a negligible increase from 93.1% to 95.3% within 15 min when the PMS dosage was further increased from 0.3 to 0.6 mM. This phenomenon could be explained as follows. When the PMS concentration was insufficient, increasing the PMS concentration would accelerate PMS activation on the catalyst surface and produce more reactive species, thus leading to rapid RhB degradation. However, when PMS exceeded the optimal dose, excessive free radicals would be generated and then react with the excessive PMS or undergo self-quenching reactions, producing some weaker oxidants and ultimately deteriorating RhB degradation. A similar study was performed by Li *et al.*^39^ on the degradation of MNZ, where they also found that the excessive PMS brought no obvious enhancement to MNZ degradation. Thus, the PMS concentration of 0.3 mM was selected as the optimal concentration.

As shown in Fig. 6e, RhB can be effectively decomposed in the pH range from 3–11 with a degradation efficiency of approximately 80% within 15 min, demonstrating that CoAl-1 could be used as an effective PMS activator for organic degradation over a wide pH range. Among them, increasing the initial pH from 3.0 to 7.0 accelerated RhB degradation efficiency from 85% to 93%. While RhB degradation efficiency instead declined from 93% to 79% with a further increase in pH from 7.0 to 11.0. The greatest removal efficiency (93.7%) was obtained at pH 7.0. This phenomenon could be explained as follows: the p*K*_a1_ and p*K*_a2_ of PMS were less than 0 and 9.4, respectively,^40,41^ and the pH_zpc_ of the CoAl-1 catalyst was 6.8. According to the pH_zpc_, the catalysts possessed a positively charged surface under acidic conditions, while the catalysts possessed a negatively charged surface under alkaline conditions. As a result, when the initial pH was increased from 3.0 to 7.0, more HSO_5_^−^ would be generated from PMS and was brought closer to the positively charged surface of the catalyst by the electrostatic gravitational forces, facilitating PMS activation thus yielding a faster RhB degradation. However, increasing the pH from 7.0 to 11.0 would enhance the electrostatic repulsion between the catalyst surface and PMS, constraining PMS activation on the catalyst surface, thus leading to lower RhB degradation. Besides, more sulfate radicals will be produced under acidic conditions, and conversely hydroxyl radicals will predominant alkaline conditions.^42^ When pH was increased from 7.0 to 11.0, the dominant species were hydroxyl radicals which were much weaker than sulfate radicals, leading to a lower degradation rate. Thus, the optimum pH for this process was 7.0.

As shown in Fig. 6f, the reaction temperature had a positive effect on RhB degradation. 93.1% of RhB was removed within 15 min at 25 °C. While 100% RhB could be degraded in 8, 6, and 4 min at 308, 318, and 328 K, respectively. The results indicated that higher temperatures could facilitate PMS activation to produce more ROS, thus accelerating RhB decomposition. RhB degradation kinetics were fitted to the pseudo-first-order kinetic model and the apparent rate constants (*k*_obs_) at 298, 308, 318, and 328 K were calculated to be 0.1687, 0.4142, 0.6279 and 0.9780 min^−1^, respectively. Based on the Arrhenius formula, the activation energy (*E*_a_) in the CoAl-1/PMS system was calculated to be 42.23 kJ mol^−1^. The *E*_a_ value was much lower than that of ordinary reactions (60–250 kJ mol^−1^),^43^ indicating that it was easier for CoAl-1 to activate PMS compared to other reported catalysts.

### 3.4 Reusability of CoAl-1

**Fig. 7:**
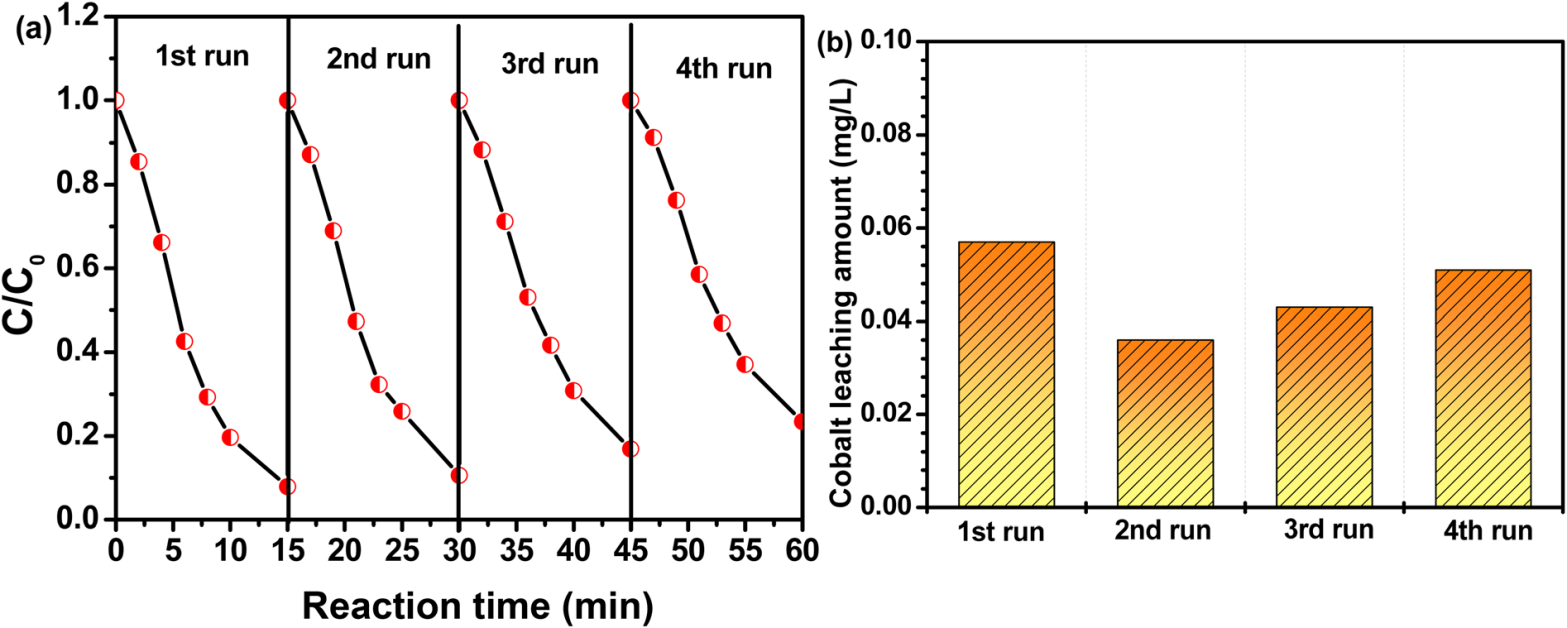
(a) Reusability of CoAl-1 for RhB degradation; (b) the amount of leached Co ions during the successive runs. [Reaction conditions: [RhB] = 80 mg L^−1^, [PMS] = 0.3 mM, [catalyst] = 100 mg L^−1^, *T* = 298 K, and pH = 7.]

The reusability of CoAl-1 was essential for its potential applications and was evaluated by reuse tests in this study. CoAl-1 particles were collected from the solution with an external magnetic field and reused under the same experimental conditions. Fig. 7a shows that the MG degradation efficiency was nearly 93.1% after 15 min in the first recycle process, and was 89.4%, 83.2% and 76.6% for the other three recycles. According to the pseudo-first-order kinetic model, the apparent rate constant (*k*) for the four recycles was estimated to be 0.1767, 0.1544, 0.1233, and 0.1022 min^−1^. In addition, the cobalt leaching amount among these cycles was tracked by ICP-MS. As shown in Fig. 7b, the cobalt leaching amount was quite low in the range of 36–57 μg L^−1^, which is far below the maximum allowable discharge quantity of cobalt (1 mg L^−1^) in water. The above results indicate that CoAl-1 exhibited desirable stability and reusability with a negligible decrease after four cycle runs. The negligible decrease of the catalyst activity might be ascribed to the accumulation of products or intermediates on the catalyst during the RhB degradation process and cobalt leaching from the catalyst in the four recycling processes.

### 3.5 Catalytic mechanism for MB removal

**Fig. 8:**
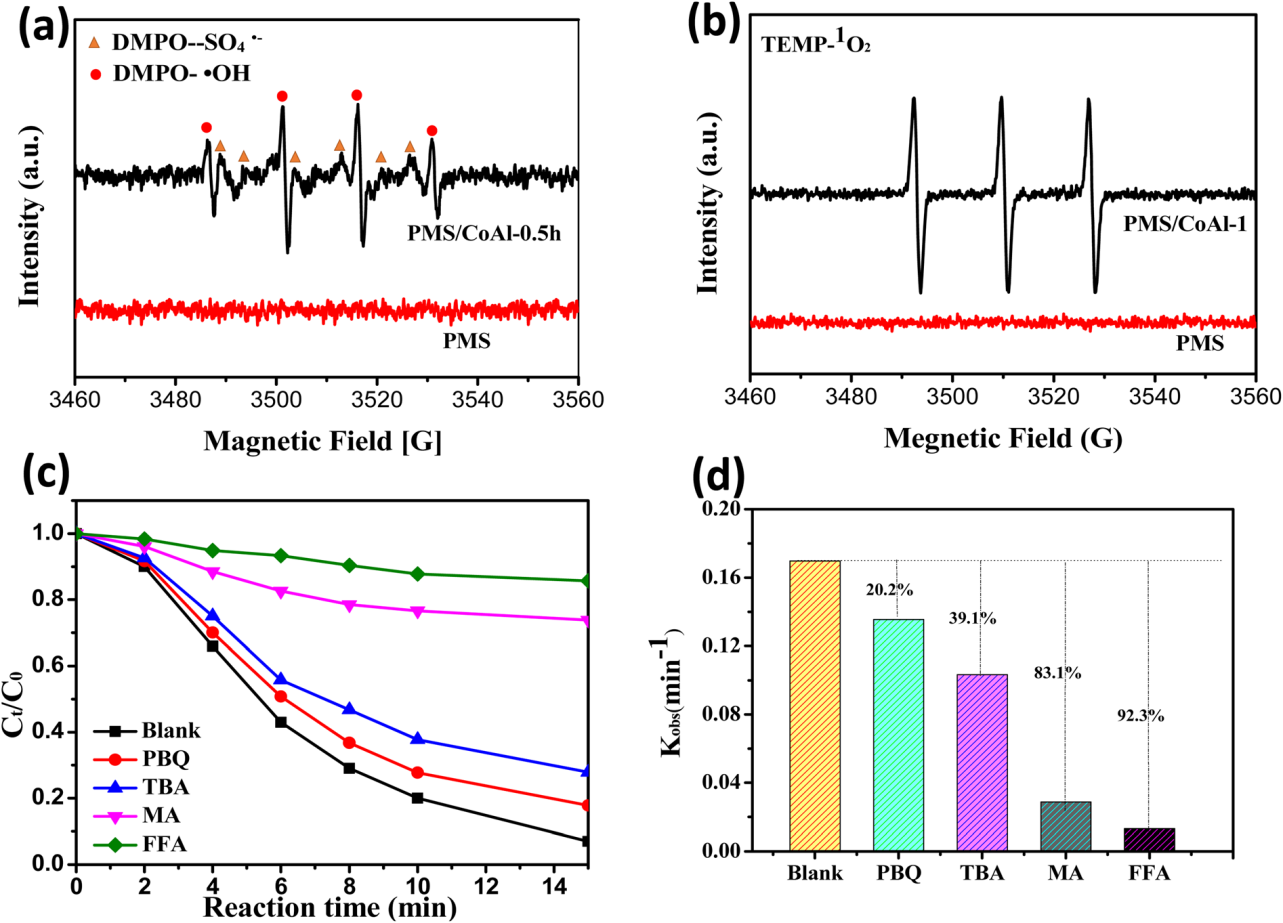
ESR spectra of (a) Temp–^1^O_2_ and (b) DMPO adducts in the (DMPO–˙OH 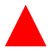, DMPO–SO_4_˙^−^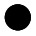); the influence of scavengers on the degradation of RhB: (c) degradation efficiency and (d) rate constant; [reaction conditions: [RhB] = 80 mg L^−1^, [catalyst] = 100 mg L^−1^, [PMS] = 0.3 mM, *T* = 298 K, pH = 7 (unadjusted), [TBA] = [MA] = [*p*-BQ] = [FFA] = 2 mM].

To shed light on the reaction mechanism, the formation of reactive oxygen species in the CoAl-1/PMS system was investigated by EPR spectra. As shown in Fig. 8a and b, the strong quadruple peaks with an intensity ratio of 1 : 2 : 2 : 1 were the characteristic peaks of the DMPO–OH adducts, while the weaker peaks were assigned to the DMPO–SO_4_ adducts. The results demonstrated the formation of ˙SO_4_^−^and ˙OH in the CoAl-1/PMS system. As previously reported, Co(ii) could activate PMS to produce ˙SO_4_^−^,^44–46^ while DMPO–SO_4_ adduct or ˙SO_4_^−^could be easily transformed into DMPO–OH or ˙OH,^47–49^ thus the signal of ˙SO_4_^−^ was weak. In addition, the significant signal of the TMP–^1^O_2_ adduct was observed, which had three characteristic peaks with equal intensity. These results confirmed the presence of ˙OH, ˙SO_4_^−^, and ^1^O_2_ in the CoAl-1/PMS system.

To further identify the contributions of ROS to RhB degradation, a series of quenching experiments were conducted, in which MeOH was used as a scavenger for ˙SO_4_^−^ and ˙OH, while TBA, *p*-BQ and FFA were chosen to quench ˙OH, ˙O_2_^−^, and ^1^O_2,_ respectively.^50^ As shown in Fig. 8c and d, *p*-BQ and TBA had little inhibitory effect on RhB degradation. When excess TBA and *p*-BQ were introduced separately into the CoAl-1/PMS system, RhB destruction efficiencies were slightly reduced from 93.1% to 72.2% and 82.2%, and the corresponding *k*_obs_ values decreased by 39.1% and 20.2% respectively within 15 min, proving that ˙OH and ˙O_2_^−^ played a negligible role in the destruction of RhB. Conversely, RhB degradation could be significantly inhibited by MeOH and FFA. When an excess of MeOH and FFA were added to the system, RhB degradation efficiency was sharply reduced to 26.2% and 14.3% from 93.1% of the blank test and the corresponding *k*_obs_ values decreased by 83.1% and 92.3%, respectively. These results indicated that ^1^O_2_ dominated RhB degradation over the CoAl-1/PMS system, while ˙OH, ˙O_2_^−^, and ˙SO_4_^−^ could mainly serve as the intermediate for the generation of ^1^O_2_ and were indirectly involved in the degradation of RhB. Furthermore, ˙SO_4_^−^ could be the dominant source of ^1^O_2_ and play an important role in RhB degradation.

Based on the above discussions, a possible mechanism for RhB degradation on CoAl-1 catalysts is proposed: on the one hand, the surface oxygen vacancy of the catalyst could serve as the active sites to mediate PMS to produce ^1^O_2_ (eqn (R1))

R1 HSO_5_^−^ + SO_5_^2−^ → HSO_4_^−^ + SO_4_^2−^ + ^1^O_2_

On the other hand, the Co^2+^/Co^3+^ redox cycle on the surface of CoAl-1 could activate PMS to provide a significant amount of ˙SO_4_^−^ and ˙SO_5_^−^ (eqn (R2) and (R3)). Subsequently, partial ˙SO_4_^−^ reacted with H_2_O or OH^−^ from the solution to form HO˙ and O_2_ (eqn (R4)). The generated O_2_ received electrons and formed O_2_˙^−^, which tended to convert into ^1^O_2_ (eqn (R5)–(R8)). Meanwhile, ˙SO_4_^−^ and HO˙ reacted with PMS to produce ˙SO_5_^−^, which could be converted into ˙SO_4_^−^ and ^1^O_2_ (eqn (R9)–(R11)). Finally, under the strong oxidation of ^1^O_2_, RhB is quickly decomposed into CO_2_ and H_2_O eqn (R12).

R2 Co^3+^ + HSO_5_^−^ → Co^2+^ + SO_5_˙^−^ + H^+^

R3 Co^2+^ + HSO_5_^−^ → Co^3+^ + ˙SO_4_^−^ + OH^−^

R4 2˙SO_4_^−^ + 2OH^−^ → 2SO_4_^2−^ + 2˙OH + O_2_

R5 O_2_ + e^−^ → ˙O_2_^−^

R6 ˙O_2_^−^ + ˙OH → ^1^O_2_

R7 ˙OH + ˙OH → H_2_O + ^1^O_2_

R8 2O_2_˙^−^ + 2H^+^ → ^1^O_2_ + H_2_O_2_

R9 ˙SO_4_^−^ + HSO_5_^−^ → ˙SO_5_^−^ + SO_4_^2−^ + 2H^+^

R10 ˙OH + HSO_5_^−^ → ˙SO_5_^−^ + H_2_O

R11 2˙SO_5_^−^ → ˙SO_4_^−^ O_2_ ˙SO_4_^−^ → 2˙SO_4_^−^ + ^1^O_2_

R12 ^1^O_2_ + RhB → CO_2_ + H_2_O + …

## 4. Conclusion

In this study, we successfully exfoliated the pristine bulk CoAl-LDHs into CoAl-LDHs nanosheets by a simple hydrothermal method in a KOH solution. The as-obtained CoAl-LDH nanosheets exhibited remarkable catalytic performance as a PMS activator for rhodamine B (RhB) degradation with the reaction rate constant (0.1687 min^−1^), which was nearly 2.22, 3.63, and 5.02 times that of CoAl-*W*, nano-Co_3_O_4_ and bulk CoAl-LDH, respectively. The superior performance was attributed to the nanosheet structure with more surface-exposed metal atoms and oxygen vacancies. A systematic analysis of the effects of reaction parameters indicated that the maximum RhB degradation was achieved under the conditions: catalyst dose 0.1 g L^−1^, PMS concentration 0.3 mM, pH 7, and temperature 298 K. Further analysis using electron paramagnetic resonance (EPR) and a series of scavenger experiments revealed that singlet oxygen (^1^O_2_) dominated RhB degradation during PMS activation, while ˙OH, ˙O_2_^−^, ˙SO_4_^−^ mainly served as intermediates for the generation of ^1^O_2_ and played an indirect role in oxidizing RhB. Overall, this study provides a simple method to fabricate LDH nanosheets used as PMS activators for enhanced water remediation.

## Conflicts of interest

There are no conflicts to declare.

## Supplementary Material

RA-013-D3RA04575G-s001

## References

[cit1] Richardson S. D., Ternes T. A. (2018). Water analysis: emerging contaminants and current issues. Anal. Chem..

[cit2] Daughton C. G., Ternes T. A. (1999). Pharmaceuticals and personal care products in the environment: agents of subtle change?. Environ. Health Perspect..

[cit3] Ahmadi M., Ghanbari F. (2019). Organic dye degradation through peroxymonosulfate catalyzed by reusable graphite felt/ferriferrous oxide: mechanism and identification of intermediates. Mater. Res. Bull..

[cit4] Li W., Wu P. X., Zhu Y., Huang Z. J., Lu Y. H., Li Y. W., Dang Z., Zhu N. W. (2015). Catalytic degradation of bisphenol A by CoMnAl mixed metal oxides catalyzed peroxymonosulfate: performance and mechanism. Chem. Eng. J..

[cit5] Cao J., Lai L., Lai B., Yao G., Chen X., Song L. (2019). Degradation of tetracycline by peroxymonosulfate activated with zero-valent iron: performance, intermediates, toxicity and mechanism. Chem. Eng. J..

[cit6] Chen B., Chen Z., Lv S. (2011). A novel magnetic biochar efficiently sorbs organic pollutants and phosphate. Bioresour. Technol..

[cit7] Wei M., Ruan Y., Luo S., Li X., Xu A., Zhang P. (2015). The facile synthesis of a magnetic OMS-2 catalyst for decomposition of organic dyes in aqueous solution with peroxymonosulfate. New J. Chem..

[cit8] Jiang B., Jing C., Yuan Y., Feng L., Liu X., Dong F., Dong B., Zhang Y. X. (2019). 2D-2D growth of NiFe LDH nanoflakes on montmorillonite for cationic and anionic dye adsorption performance. J. Colloid Interface Sci..

[cit9] Tian M., Thind S. S., Dondapti J. S., Li X., Chen A. (2018). Electrochemical oxidation of 4- chlorophenol for wastewater treatment using highly active UV treated TiO_2_ nanotubes. Chemosphere.

[cit10] Lee J., von Gunten U., Kim J. H. (2020). Persulfate-based advanced oxidation: critical assessment of opportunities and roadblocks. Environ. Sci. Technol..

[cit11] Wang J., Wang S. (2018). Activation of persulfate (PS) and peroxymonosulfate (PMS) and application for the degradation of emerging contaminants. Chem. Eng. J..

[cit12] Ji Y., Dong C., Kong D., Lu J., Zhou Q. (2015). Heat-activated persulfate oxidation of atrazine: implications for remediation of groundwater contaminated by herbicides. Chem. Eng. J..

[cit13] Rehman F., Sayed M., Khan J. A., Shah N. S., Khan H. M., Dionysiou D. D. (2018). Oxidative removal of brilliant green by UV/S_2_O_8_^2-^, UV/HSO_5_^-^ and UV/H_2_O_2_ processes in aqueous media: a comparative study. J. Hazard. Mater..

[cit14] Zhao Q., Mao Q., Zhou Y., Wei J., Liu X., Yang J., Luo L., Zhang J., Chen H., Chen H., Tang L. (2017). Metal-free carbon materials-catalyzed sulfate radical-based advanced oxidation processes: a review on heterogeneous catalysts and applications. Chemosphere.

[cit15] Kohantorabi M., Hosseinifard M., Kazemzadeh A. (2020). Catalytic activity of a magnetic Fe_2_O_3_@CoFe_2_O_4_ nanocomposite in peroxymonosulfate activation for norfloxacin removal. New J. Chem..

[cit16] Tan C., Lu X., Cui X., Jian X., Hu Z., Dong Y., Liu X., Huang J., Deng L. (2019). Novel activation of peroxymonosulfate by an easily recyclable VC@Fe_3_O_4_ nanoparticles for enhanced degradation of sulfadiazine. Chem. Eng. J..

[cit17] Xian G., Zhang G., Chang H., Zhang Y., Zou Z., Li X. (2019). Heterogeneous activation of persulfate by Co_3_O_4_-CeO_2_ catalyst for diclofenac removal. J. Environ. Manage..

[cit18] Li Z., Liu K., Fan K., Yang Y., Shao M., Wei M., Duan X. (2019). Active-oxygen enhanced homogeneous nucleation of lithium metal on ultrathin layered double hydroxide. Angew. Chem..

[cit19] Wang Q., Chen L. F., Guan S. L., Zhang X., Wang B., Cao X. Z., Yu Z., He Y. F., Evans D. G., Feng J. T., Li D. Q. (2018). Ultrathin and Vacancy-Rich CoAl-Layered Double Hydroxide/Graphite Oxide Catalysts: Promotional Effect of Cobalt Vacancies and Oxygen Vacancies in Alcohol Oxidation. ACS Catal..

[cit20] Huang L., Megías-Sayago C., Bingre R., Zheng Q., Wang Q., Louis B. (2019). Catalytic performance of layered double hydroxides (LDHs) derived materials in gas-solid and liquid-solid phase reactions. ChemCatChem.

[cit21] Fan G., Li F., Evans D. G., Duan X. (2014). Catalytic applications of layered double hydroxides: recent advances and perspectives. Chem. Soc. Rev..

[cit22] Sun J. A., Wang L. X., Wang Y. G., Lv W. Y., Yao Y. Y. (2022). Activation of peroxymonosulfate by MgCoAl layered double hydroxide: potential enhancement effects of catalyst morphology and coexisting anions. Chemosphere.

[cit23] Kohantorabi M., Moussavi G., Giannakis S. (2021). A review of the innovations in metal and carbon-based catalysts explored for heterogeneous peroxymonosulfate (PMS) activation, with focus on radical vs. non-radical degradation pathways of organic contaminants. Chem. Eng. J..

[cit24] Zong Y., Li K., Tian R., Lin Y., Lu C. (2018). Highly dispersed layered double oxide hollow spheres with sufficient active sites for adsorption of methyl blue. Nanoscale.

[cit25] Ma R. Z., Liu Z. P., Li L., Iyi N., Sasaki T. (2006). Exfoliating layered double hydroxides in formamide: a method to obtain positively charged nanosheets. J. Mater. Chem..

[cit26] Yu J. F., Liu J. J., Clearfield A., Sims J. E., Speiegle M. T., Suib S. L., Sun L. Y. (2016). Synthesis of Layered Double Hydroxide Single-Layer Nanosheets in Formamide. Inorg. Chem..

[cit27] Guo R. N., Zhu Y. L., Cheng X. W., Li J. J., Crittenden J. C. (2020). Efficient degradation of lomefloxacin by Co-Cu-LDH activating peroxymonosulfate process: optimization, dynamics, degradation pathway and mechanism. J. Hazard. Mater..

[cit28] Zhang Y. P., Xu H. F., Lu S. (2021). Preparation and application of layered double hydroxide nanosheets. RSC Adv..

[cit29] Chen B., Zhang Z., Kim S. K., Lee S. G., Lee J. W., Kim W. Y., Yong K. J. (2018). Ostwald Ripening Driven Exfoliation to Ultrathin Layered Double Hydroxides Nanosheets for Enhanced Oxygen Evolution Reaction. ACS Appl. Mater. Interfaces.

[cit30] Wang Y. Y., Zhang Y. Q., Liu Z. J., Xie C., Feng S., Liu D. D., Shao M. F., Wang S. Y. (2017). Layered Double Hydroxide Nanosheets with Multiple Vacancies Obtained by Dry Exfoliation as Highly Efficient Oxygen Evolution Electrocatalysts. Angew. Chem., Int. Ed..

[cit31] Zhang X., Zhao Y. F., Zhao Y. X., Shi R., Waterhouse G. I. N., Zhang T. R. (2019). A Simple Synthetic Strategy toward Defect-Rich Porous Monolayer NiFe-Layered Double Hydroxide Nanosheets for Efficient Electrocatalytic Water Oxidation. Adv. Energy Mater..

[cit32] Liu Z., Teng L., Ma L. F., Liu Y., Zhang X. Y., Xue J. L., Ikram M., Ullah M., Li L., Shi K. Y. (2019). Porous 3D flower-like CoAl-LDH nanocomposite with excellent performance for NO_2_ detection at room temperature. RSC Adv..

[cit33] Jiang S. D., Song L., Zeng W. R., Huang Z. Q., Zhan J., Stec A. A., Hull T. R., Hu Y., Zhao W. (2015). Self-Assembly Fabrication of Hollow Mesoporous Silica@CoAl Layered Double Hydroxide@Graphene and Application in Toxic Effluents Elimination. ACS Appl. Mater. Interfaces.

[cit34] Zhang M., Liu Y., Liu B., Chen Z., Xu H., Yan K. (2020). Trimetallic NiCoFe-layered double hydroxides nanosheets efficient for oxygen evolution and highly selective oxidation of biomass-derived 5-hydroxymethylfurfural. ACS Catal..

[cit35] Li H., Wang H., Gao Q., Han B., Xia K., Zhou C. (2020). Hierarchical flower-like Co_2_TiO_4_ nanosheets with unique structural and compositional advantages to boost peroxymonosulfate activation for degradation of organic pollutants. J. Mater. Chem. A.

[cit36] Oh W. D., Lua S. K., Dong Z., Lim T. T. (2015). Performance of magnetic activated carbon composite as peroxymonosulfate activator and regenerable adsorbent via sulfate radical-mediated oxidation processes. J. Hazard. Mater..

[cit37] Li Z. D., Liu D. F., Zhao Y. X., Li S. R., Wei X. C., Meng F. S., Huang W. L., Lei Z. F. (2019). Singlet oxygen dominated peroxymonosulfate activation by CuO-CeO_2_ for organic pollutants degradation: performance and mechanism. Chemosphere.

[cit38] Yan J., Li J., Peng J., Zhang H., Zhang Y., Lai B. (2019). Efficient degradation of sulfamethoxazole by the CuO@Al_2_O_3_ (EPC) coupled PMS system: optimization, degradation pathways and toxicity evaluation. Chem. Eng. J..

[cit39] Li H. T., Gao Q., Wang G. H., Han B., Xia K. H., Wu J. P., Zhou C. G., Dong J. (2021). Postsynthetic incorporation of catalytically inert Al into Co_3_O_4_ for peroxymonosulfate activation and insight into the boosted catalytic performance. Chem. Eng. J..

[cit40] Tan C., Lu X., Cui X., Jian X., Hu Z., Dong Y., Liu X., Huang J., Deng L. (2019). Novel activation of peroxymonosulfate by an easily recyclable VC@Fe_3_O_4_ nanoparticles for enhanced degradation of sulfadiazine. Chem. Eng. J..

[cit41] Li H., Gao Q., Wang G., Han B., Xia K., Zhou C. (2020). Architecturing CoTiO_3_ overlayer on nanosheets-assembled hierarchical TiO_2_ nanospheres as a highly active and robust catalyst for peroxymonosulfate activation and metronidazole degradation. Chem. Eng. J..

[cit42] Ding M., Chen W., Xu H., Shen Z., Lin T., Hu K., Lu Ch., Xie Z. (2020). Novel α-Fe_2_O_3_/MXene nanocomposite as heterogeneous activator of peroxymonosulfate for the degradation of salicylic acid. J. Hazard. Mater..

[cit43] Li H., Gao Q., Wang G., Han B., Xia K., Zhou C. (2021). Fabricating yolk–shell structured CoTiO_3_@ Co_3_O_4_ nanoreactor via a simple self-template method toward high performance peroxymonosulfate activation and organic pollutant degradation. Appl. Surf. Sci..

[cit44] Kohantorabi M., Moussavi G., Giannakis S. (2020). A review of the innovations in metaland carbon-based catalysts explored for heterogeneous peroxymonosulfate (PMS) activation, with focus on radical vs. non-radical degradation pathways of organic contaminants. Chem. Eng. J..

[cit45] Mian M. M., Liu G., Fu B., Song Y. (2019). Facile synthesis of sludge-derived MnO_x_-Nbiochar as an efficient catalyst for peroxymonosulfate activation. Appl. Catal., B.

[cit46] Zhong Q. F., Lin Q. T., Huang R. L., Fu H. Y., Zhang X. F., Luo H. Y., Xiao R. B. (2020). Oxidative degradation of tetracycline using persulfate activated by N and Cu codoped biochar. Chem. Eng. J..

[cit47] Yang S. S., Zhang S. X., Li X., Du Y. X., Xing Y. X., Xu Q., H Wang Z., Li L., Zhua X. T. (2022). One-step pyrolysis for the preparation of sulfur doped biochar loaded with iron nanoparticles as an effective peroxymonosulfate activator for RhB degradation. New J. Chem..

[cit48] Ren Y., Lin L., Ma J., Yang J., Feng J., Fan Z. (2015). Sulfate radicals induced from peroxymonosulfate by magnetic ferrospinel MFe_2_O_4_ (M = Co, Cu, Mn, and Zn) as heterogeneous catalysts in the water. Appl. Catal., B.

[cit49] Wu L., Zhang Q., Hong J., Dong Z., Wang J. (2019). Degradation of bisphenol A by persulfate activation via oxygen vacancy-rich CoFe_2_O_4-x_. Chemosphere.

[cit50] Rao L. J., Yang Y. F., Chen L. K., Liu X. D., Chen H. X., Yao Y. Y., Wang W. T. (2020). Highly efficient removal of organic pollutants via a green catalytic oxidation system based on sodium metaborate and peroxymonosulfate. Chemosphere.

