# Supplementary material for: Facile fabrication of CoAl-LDH nanosheets for efficient rhodamine B degradation via peroxymonosulfate activation
Source: RSC Adv. 2023 Oct 10;13(42):29695–705. doi: 10.1039/d3ra04575g (PMC10563443; doi:10.1039/d3ra04575g)
Supplement: RA-013-D3RA04575G-s001 [file RA-013-D3RA04575G-s001.pdf]

Electronic Supplementary Material (ESI) for RSC Advances.
This journal is © The Royal Society of Chemistry 2023

Supporting Information

Facile fabrication of CoAl-LDH nanosheets for efficient rhodamine B degradation through peroxymonosulfate activation

HUi Fui,*[a] Shuming Gao, [a] Yiping Huang, [a] Xinran Ma [a]

[a] School of Chemical and Environmental Engineering, Wuhan Polytechnic University, Wuhan 430023,PR China

*Corresponding author: HUi Fui: Feihui509@163.com

Text S1. Reuse test

To collect and separate the products of the CoAl-1/PMS/ RhB system, the reaction volume was increased by 100 times to 2 L of reaction solution containing 100 mg catalyst, 80 mg RhB, 0.3 mmol. After the 15 min, the solution was quenched with $Na_2S_2O_3$ solution ($Na_2S_2O_3$: PMS = 30:1 in molar concentration). The quenched samples solution was placed in external magnet field for 2 h and then most of the supernatants were removed. The resulting suspensions were centrifuged to separate the solid and liquid. The solid products were collected and washed with DI and ethanol for three times before the next run.

To track Cobalt leaching amount among the reuse tests, sample was withdrawn and quenched with $Na_2S_2O_3$ solution ($Na_2S_2O_3$: PMS = 30:1 in molar concentration) after each run. Then, the quenched samples were centrifuged to separate the solid and liquid. The supernatants were collected and analyzed by Inductively Coupled Plasma-Mass Spectrometry (ICP–MS, Optima 5300 DV, PerkinElmer, America).

Text S2. Determine the pH point of zero charge (pHzpc)

Typically, 50 mL of KCl 0.1 M solution at six different pH (3, 5, 7, 9, 11) and 0.01 g of CoAl-1 nanoparticles were applied to the determination of pHzpc. Then place the prepared solutions on the shaker for 24 h and measured the final pH after 24 h $\Delta pH = pH_{final} - pH_{initial}$ was calculated and its curve plotted as the X = initial pH and Y = ΔpH. The intersection of the curve with the X-axis is equal to pHzpc.

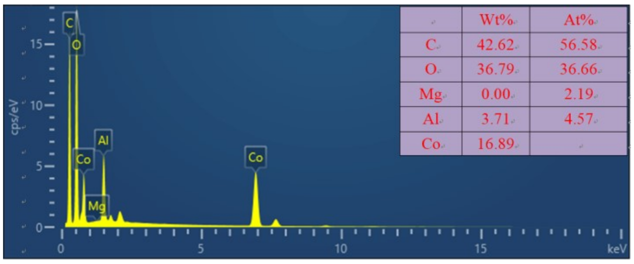

Figure S1. EDS spectra for CoAl-1 nanosheets:

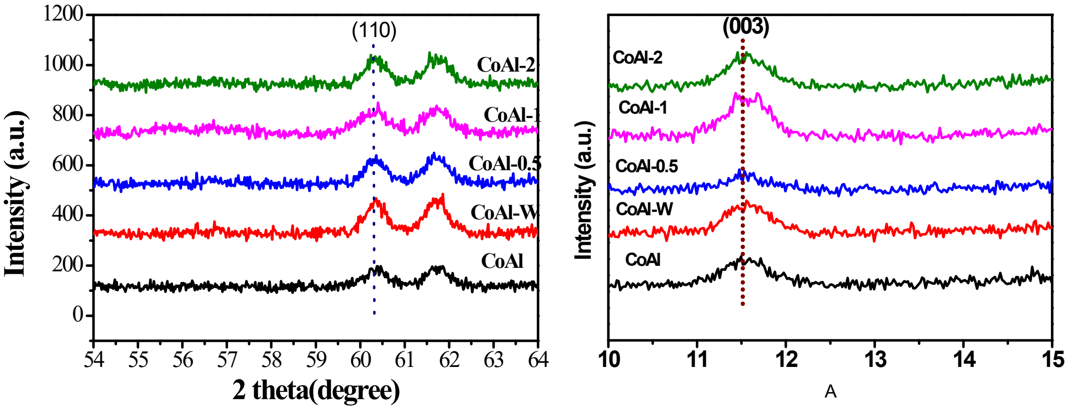

Figure S2. XRD patterns for ZnAl-LDH nanosheets and X-ZnAl-LDH nanosheets (X = 0.25%, 0.5%, 1%, 3%): (a) Expanded view from 2θ = 54-64° over the (110) diffraction peak; (b) Expanded view from 2θ = 10-15° over the (003) diffraction peak

Table S1. Unit cell parameters for the CoAl-LDH and CoAl-1 nanosheets: determined from XRD analyses.

sample	$2\theta_{(003)}$	$d_{(003)}$[Å]	$2\theta_{(110)}$	$d_{(110)}$[Å]	a[Å]	c[Å]	Strain(%)
CoAl	**11.64**	**7.596**	**60.20**	**1.536**	**3.072**	**22.788**	
CoAL-W	**11.66**	**7.583**	**60.32**	**1.533**	**3.066**	**22.749**	
CoAL-0.5	**11.70**	**7.558**	**60.34**	**1.532**	**3.064**	**22.674**	
CoAL-1	**11.78**	**7.506**	**60.38**	**1.533**	**3.066**	**22.518**	
CoAL-2	**11.76**	**7.520**	**60.46**	**1.530**	**3.060**	**22.560**	

The lattice parameters *a* and *b* were calculated using the equation: $a = b = 2d(110)$. This was then used to estimate the degree of strain around the *ab*-plane in the LDH nanosheets.[9].The lattice parameter c was calculated using the equation $c = 3d(003)$.

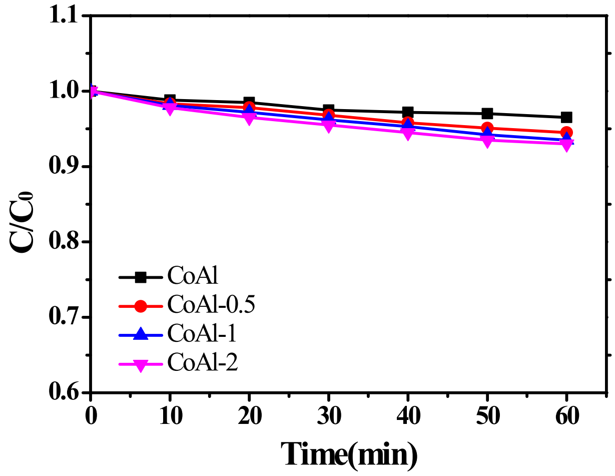

Fig. S3 Adsorption experiments over different samples. Reaction conditions: V=100mL, [RhB]=80mg/L, [catalyst]=0.1 g/L, T=298 K, pH=7.

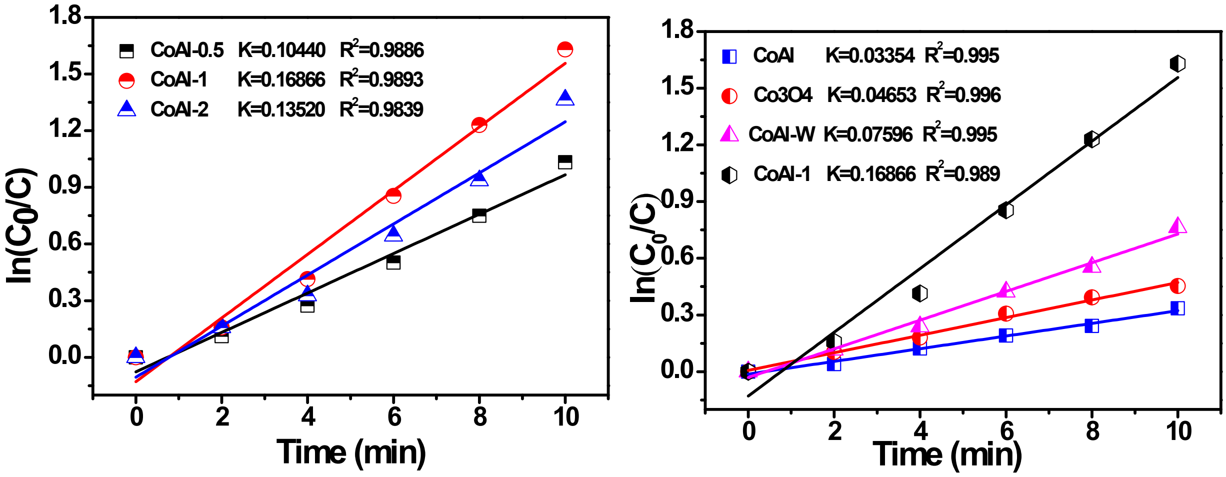

Fig. S4 Kinetic **curves** of RhB removal with different catalysts. (a) different exfoliated CoAl-LDH, (b) bulk CoAl-LDH , Co_3O_4, CoAl-W and CoAl-1. Reaction conditions: V=100mL, [RhB]=80mg/L, [catalyst]=0.1 g/L, [PMS]= 0.1 g/L, T=298 K, pH=7.

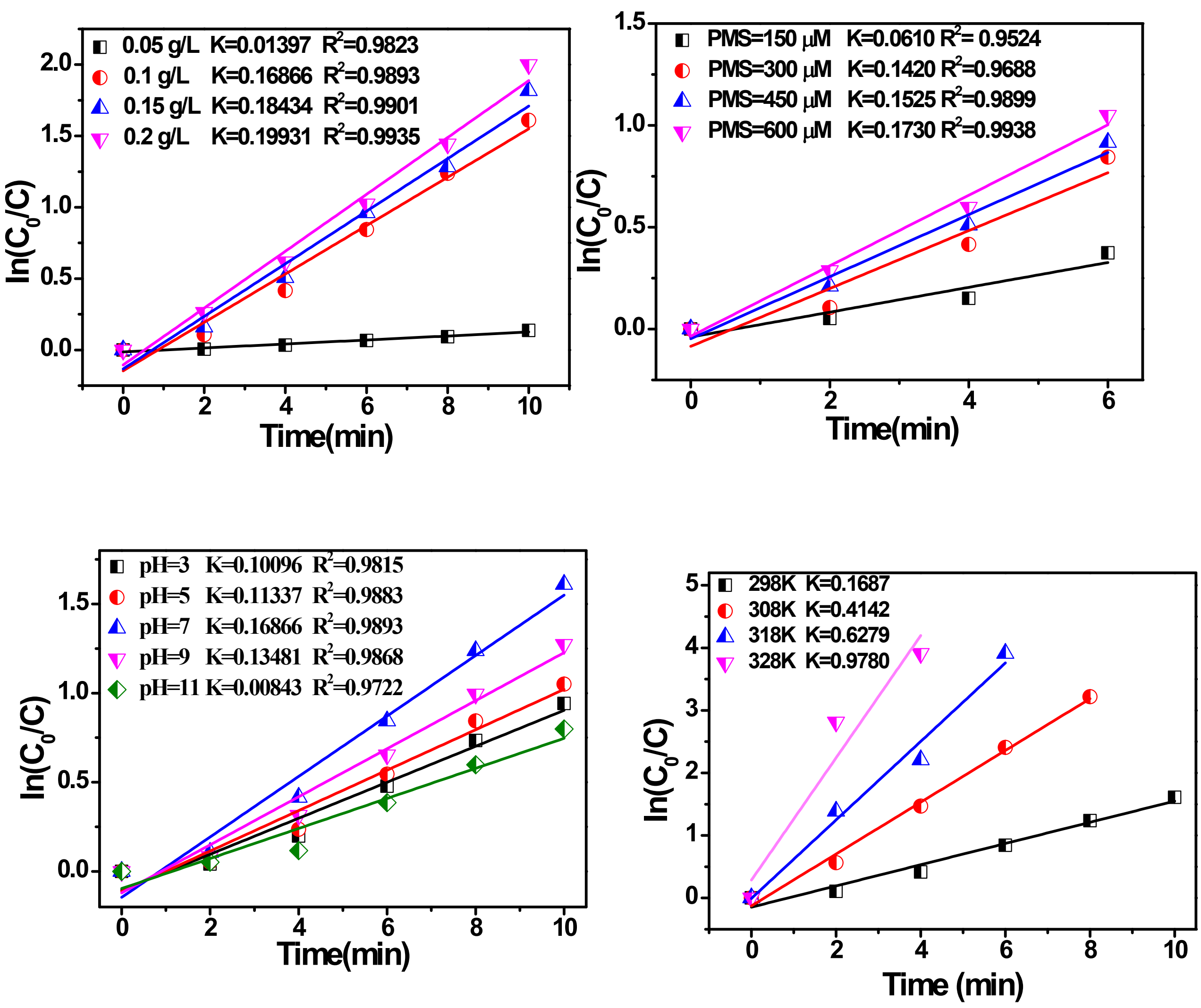

Fig. S5 **Kinetic curves of RhB removal at different reaction conditions. (a)** catalyst dose, (b) PMS concentration, (c) initial solution pH, (d) solution temperature. Reaction conditions: V=100mL, [RhB] =80 mg L^{-1}, [PMS]= 0.3mM,[catalyst] = 100 mg L^{-1}, T =298 K and pH =7.

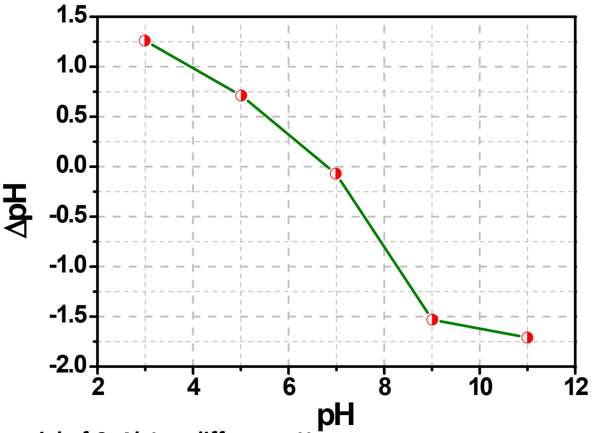

Fig. S6 **Zeta potential of CoAl-1 at different pH.**

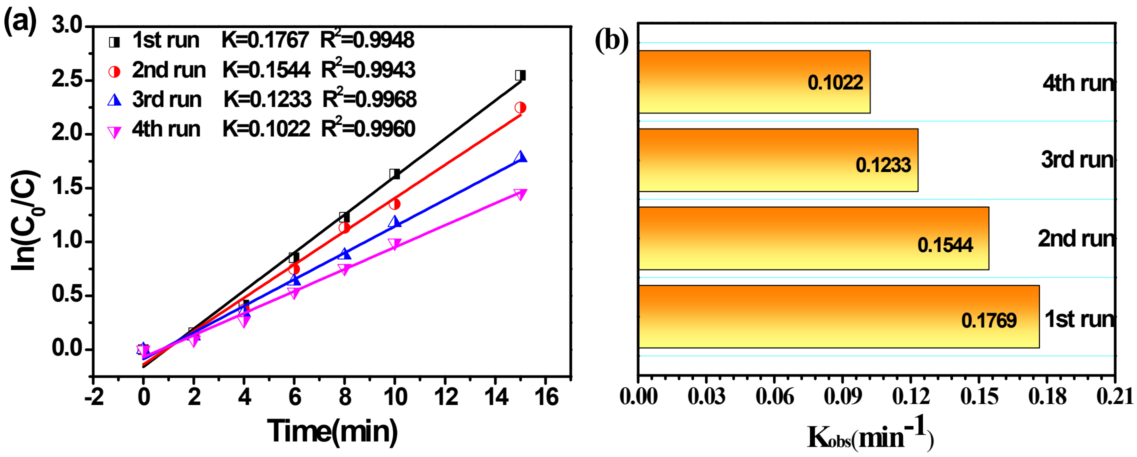

Fig. S7 (a) Kinetic **curves** of RhB removal in multiple runs with recycled CoAl-1, (b) rate constant of RhB removal for multiple runs. Reaction conditions: V=100mL, [RhB]=80mg/L, [catalyst]=0.1 g/L, [PMS]= 0.1 g/L, T=298 K, pH=7.

Table S2 Comparison with reported works of other catalyst systems

Catalyst	Reaction conditions	Time (min)	Removal efficiency	Ref
**Co_1Fe_1-LDHs nanosheets**	[catalyst] = 0.05 g/L, [PMS] = 0.25 mM, **[CIP]** = 20 mg/L	12	86.9%	1
**MgCoAl-LDH nanoscrolls**	[MB] = 50 µM, [PMS] = 1 mM, [catalyst] = 0.02 g/L	40	100%	2
**Co_2Cu_1-LDH nanosheets**	[PMS] = 0.15 g/L, [catalyst] = 0.04 g/ L [LOM] = 10 mg/L	30	96.2 %	3
**Fe_3O_4@CoFe-LDH nanocomposite**	[AO7] = 40 mg/L, [catalyst] = 50 mg/L, [PMS] = 0.12 mM	15	95.1%	4
**CoAl-LDHs nanosheets**	[RhB] = 80 mg/L, [catalyst] = 0.1 g/L, [PMS] = 0.3 mM	15	93.1%	**This work**

References

1. Z. Y. Yang, X. Li , Y. Z. Huang , Y. W. Chen , A. Q. Wang , Y. C. Wang , C. H. Li , Z. F. Hu, K. Yan, Facile synthesis of cobalt-iron layered double hydroxides nanosheets for direct activation of peroxymonosulfate (PMS) during degradation of fluoroquinolones antibiotics, Journal of Cleaner Production, 2021, 310, 127584-127596.

2. J. A. Sun, L. X. Wang, Y. G. Wang, W. Y. Lv , Y. Y. Yao; Activation of peroxymonosulfate by MgCoAl layered double hydroxide: Potential enhancement effects of catalyst morphology and coexisting anions, *2022, Chemosphere, 286 , 131640-131650.*

3. R. N. Guo, Y. L. Zhu, X. W. Cheng, J. J. Li, J. C. Crittenden, Efficient degradation of lomefloxacin by Co-Cu-LDH activating peroxymonosulfate process: Optimization, dynamics, degradation pathway and mechanism, J. Hazard Mater., 2020, 399, 122966-122980.

4. D. P. Sun, C. Li, S. S. Lu, Q. F. Yang and C. Q. He, Magnetic Fe_3O_4@CoFe-LDH nanocomposite heterogeneously activated peroxymonosulfate for degradation of **azo-dye AO7**, RSC Adv., 2021, 11, 20258-20267.
